# Development of a polymerase chain reaction assay for the rapid detection of the oral pathogenic bacterium, *Selenomonas noxia*

**DOI:** 10.1186/s12903-015-0071-1

**Published:** 2015-08-14

**Authors:** Patricia Cruz, Arthuro M. Mehretu, Mark P. Buttner, Theresa Trice, Katherine M. Howard

**Affiliations:** Department of Environmental and Occupational Health, School of Community Health Sciences, University of Nevada Las Vegas, 4504 S. Maryland Parkway, Box 3064, Las Vegas, NV 89154-3064 USA; MPH Program, Epidemiology & Biostatistics Concentration, Department of Environmental and Occupational Health, School of Community Health Sciences, University of Nevada, Las Vegas, NV USA; Southern Nevada Health District, Las Vegas, NV USA; Department of Biomedical Sciences, School of Dental Medicine, University of Nevada, Las Vegas, NV USA

**Keywords:** Bacteria, Periodontal health and disease, *Selenomonas noxia*, PCR

## Abstract

**Background:**

In recent studies, periodontal health has been linked to being overweight and/or obese. Among common oral bacteria, *Selenomonas noxia* has been implicated in converting periodontal health to disease, and *Selenomonas* species have also been found in gastric ulcers. The objective of this study was to develop and validate a quantitative polymerase chain reaction (qPCR) assay for the specific and rapid detection of *S. noxia*.

**Methods:**

Two oligonucleotide primer pairs and one probe were designed and tested to determine optimal amplification signal with three strains of *S. noxia*. The PCR assay was tested against fourteen non-target organisms, including closely related oral Selenomonads, one phylogenetically closely related bacterium, and two commonly isolated oral bacteria.

**Results:**

One of the primer sets was more sensitive at detecting the target organism and was selected for optimization and validation experiments. The designed primers and probe amplified the target organism with 100 % specificity. PCR inhibition was observed with an internal positive control, and inhibition was resolved by diluting the DNA extract.

**Conclusions:**

The qPCR assay designed in this study can be used to specifically detect *S. noxia* in the clinical setting and in future research involving the enhanced detection of *S. noxia*. The assay can also be used in epidemiological studies for understanding the role of *S. noxia* in disease processes including, but not limited to, oral health and obesity of infectious origin.

## Background

The genus *Selenomonas* was first described in 1683 by Antony Van Leeuwenhoek as a crescent-shaped bacterium from an oral sample [1]. *Selenomonas noxia* is a bacterium that colonizes the human oral cavity, and has been repeatedly associated with periodontal disease [2–4]. This species is composed of obligately anaerobic, motile, non-spore-forming, gram negative rods [5]. *S. noxia* was among the five new species of the genus *Selenomonas*, Phylum Firmicutes, characterized for the first time by Moore *et al*. in 1987 [5]. Periodontal diseases are probably one of the most common bacterial infections in humans. Only a few of the several hundred species of microorganisms that have been identified within the gingival crevice and the periodontal pocket are thought to play a significant role in initiation and progression of the disease [6], and *S. noxia* was among the new organisms added as a putative periodontal pathogen [7]. Very little literature is available on the pathogenecity of *S. noxia*, but the genus *Selenomonas* has been found in higher proportions compared to other oral bacteria in cases of generalized aggressive periodontitis (GAP). In addition, *S. noxia* has been detected using culture and DNA-based techniques in both chronic periodontitis (CP) and GAP lesions [8, 9]. In a study conducted by Kumar *et al.* [10], disease-associated samples were collected from the four deepest sites in subjects with established periodontitis, and the most numerous species by 16S clonal analysis belonged to the genera *Selenomonas*, *Streptococcus*, *Veillonella*, *Campylobacter*, and *Peptostreptococcus*. While *Selenomonas* oral clones were not associated with disease [8], other studies have suggested that *S. noxia* is among the principal species associated with sites converting periodontal health to periodontal disease [3, 11]. Furthermore, in a recent report by Andersen *et al.* [12], a *Helicobacter*-Like Organism (HLO) in the histological section from a human gastric ulcer was found to be a *Selenomonas* species. The literature and studies conducted to date do not give precise data on the overall prevalence of *S. noxia* in healthy individuals.

Molecular techniques have provided a good basis in identifying the role of *Selenomonas* species as periodontal health indicators [8, 13]. In a study using oligonucleotide probes targeting the majority of all oral isolates to explore the spatial distribution of *Selenomonas* species in subgingival biofilms, a relatively low prevalence of *Selenomonas* was shown in GAP and CP patients. Nevertheless, a fluorescence *in-situ* hybridization (FISH) analysis of the same biofilm showed that *Selenomonas* made a relevant contribution to the structural organization of the biofilms [9].

In addition to the role of *S. noxia* in oral health, a study by Goodson *et al.* [14] implied that *S. noxia* can be associated with obesity. The study demonstrated that 98.4 % of the overweight individuals were correctly identified by the presence of the single bacterium, *S. noxia*. This finding provided a clue to better understand the association and/or presence of *S. noxia* in the oral cavity and the development of obesity. Recently, there has been increasing interest in understanding the relationship between human microbial diversity and being overweight or obese. Considering the likelihood that periodontal disease may contribute to the development of obesity, the role of the oral microbiome in obesity has been gaining more attention [14]. The mechanism by which oral bacteria contribute to the development of obesity is explained in at least three ways: increasing metabolic efficiency, increasing appetite, and/or redirecting energy metabolism [14].

The increasing clinical and epidemiological importance of *S. noxia* necessitates the development of a rapid detection method. Until now, detection methods for *S. noxia* have been limited to culture and DNA hybridization tests. Culture analysis of *Selenomonas* spp. is not common in the clinical microbiological laboratory and may take time because it is a fastidious obligately anaerobic bacterium. With the anaerobic culture technique, the discrepancy between salivary recovery and subgingival presence has been significant, which makes this approach unsuitable for use in the microbial diagnosis of periodontitis patients [15]. Using the polymerase chain reaction (PCR) detection technique with species specific oligonucleotide primers and probes can enhance the rapid detection of *S. noxia*. The quantitative PCR (qPCR) method combines PCR chemistry with fluorescent probe detection of amplified product in the same reaction vessel, and is completed in two hours or less. The objective of this study was to develop and validate a qPCR assay for the specific detection of *S. noxia*. Enhanced detection of *Selenomonas noxia* in the oral microflora is the first step in elucidating its involvement in obesity and human disease.

## Methods

### Test organisms

Three different strains of *S. noxia* were obtained from the American Type Culture Collection (ATCC, Manassas, VA) and used to evaluate the designed PCR protocol (Table 1). Fourteen non-target organisms, including other closely related oral Selenomonads, one phylogenetically closely related bacterium, and two commonly found oral bacteria*,* were obtained from ATCC and used to test the specificity of the designed primers and probe (Table 1).

**Table 1:** Test organisms

Bacterial species	ATCC #
*Bacillus cereus*	14579
*Candida albicans*	14053
*Centipeda periodontii*	35019
*Klebsiella pneumoniae*	4352
*Lactobacillus acidophilus*	3456
*Pectinatus cervisiiphilus*	29359
*Pseudomonas aeruginosa*	27853
*Selenomonas artemidis*	43528
*Selenomonas dianae*	43527
*Selenomonas flueggei*	43531
*Selenomonas infelix*	43532
*Selenomonas noxia*	43541
*Selenomonas noxia*	51893
*Selenomonas noxia*	700225
*Selenomonas sputigena*	35185
*Staphylococcus aureus*	25923
*Streptococcus mutans*	25175

### DNA extraction

Microbial DNA was extracted using the QIAamp® DNA Mini Kit (Qiagen, Valencia, CA) according to the manufacturer’s instructions, with the following changes; 100 μl of sample volume was used for extraction and the final elution volume was 200 μl. Selected samples were extracted with the UltraClean® Soil DNA Isolation Kit (MoBio, Carlsbad, CA). The DNA extracts were stored at −70 °C until ready to use.

The presence and amount of DNA in each sample was measured with a Spectronic™ GENESYS 10 Bio UV-Visible spectrophotometer using the nanoCell accessory-0.2 mm pathlength (Thermo Electron Corporation, Madison, WI) to analyze sub-microliter DNA samples in solution. The assay was performed with 1.5 μl of test sample, after being zeroed with TRIS-EDTA (TE) buffer (pH 7.4). DNA/Protein concentration mode (Absorbance at 260 and 280 nm with 320 nm correction) was used for measurement. The DNA concentrations in *S. noxia* (ATCC 43541, ATCC 700225, and ATCC 51893) were between 4.2 and 11.7 ng/μl. Concentrations ranged from 4.2 to 150.8 ng/μl in the non-target samples (data not shown). DNA template concentrations used for PCR varied, but were sufficient to obtain a positive result in a presence/absence test, based on the sensitivity of the assay (583 fg/reaction).

### Primer and probe design

The nucleotide coding region sequence and entire genome sequence data of *S. noxia* were retrieved from the National Center for Biotechnology Information, NCBI (http://www.ncbi.nlm.nih.gov/Genomes/). Sequence assembly and alignment were compared *in-silico* against all available sequences on-line with the Basic Local Alignment Search Tool algorithm (BLAST, NCBI).

The 16S ribosomal RNA (1491 base pair) of *S. noxia* (ATCC 43541) was selected to design specific primers and probes by comparing the V8 region of this gene (Table 2). This region is highly conserved among members of the *Selenomonas* genus, but more variable among *Selenomonas* species. TaqMan® primers and probes were designed and analyzed using the Primer Express software version 3 (Life Technologies [Applied Biosystems], Foster City, CA). Selected primers were screened against formation of secondary structures, including formation of hairpin structures, and self-and cross-dimers. The primer length, melting temperature (T_m_), G-C ratio and other factors were set as defaults on the Primer Express software.

**Table 2:** Multiple alignments of V8 regions of the 16S rRNA gene from *Selenomonas* species. Numbering used according to *Escherichia coli* 16S rRNA gene [31]

Species	ATCC #	V8 region (bp 1268 to 1296)
*S. noxia*	43541	CAGAGGGCAGCGAGAGA-GTGATCTTAAGC
*S. artemidis*	43528	.....A.........-.CCC.C..GGGC....
*S. flueggei*	43531	.......A.......-..GC.C..G.CGG…
*S. infelix*	43532	.....A.........-.CCC.C..GGGC....
*S. sputigena*	35185	.....A................-.C..........
*S. dianae*	43527	.....A.........-.CCC.C..GGGC....

Two primer pairs and one probe were selected for testing; i) Primer Set 1: Forward primer- SNF1, TCTGGGCTACACACGTACTACAATG (25 bp) and Reverse primer- SNR1, GCCTGCAATCCGAACTGAGA (20 bp), ii) Primer set 2: Forward primer- SNF2, GCATGTAAAGATGGGCACTCAA (22 bp) and Reverse primer- SNR2, CCGAACTGAGAAACGGTTTTTG (22 bp); with amplicon lengths of 97 and 175, respectively. The probe (SnP) selected for both primer sets was labeled at the 5’-end with the reporter dye 6-carboxyfluorescein (FAM) and the 3’-end with the reporter dye tetramethyl-6-carboxyrhodamine (TAMRA), SnP, [6 ~ FAM]CAGAGGGCAGCGAGAGAGTGATCTTAAGC [TAMRA]. The designed primers and probe were obtained from Eurofins MWG Operon (Huntsville, AL). Both primer sets were tested with DNA from two *S. noxia* reference strains (ATCC 43541 and 51893) to determine the optimal amplification signal.

### Primer selection and PCR optimization

Nine different combinations, between 0.2 μM and 0.9 μM, of the forward and reverse primers and five different concentrations of probe ranging from 0.05 μM to 0.25 μM were tested with DNA (600 pg) from *S. noxia* reference strain ATCC 51893 to determine the optimal amplification signal. The PCR cycling parameters were as follows: initial incubation step of 50 °C for 2 min, denaturation of the template DNA at 95 °C for 10 min, followed by 40 cycles at 95 °C for 15 sec and 60 °C for 1 min. PCR conditions were: 1X TaqMan universal PCR master mix containing AmpErase® UNG (uracil-N-glycosylase), AmpliTaq Gold DNA polymerase, deoxynucleoside triphosphates, passive internal reference [ROX™ dye], and optimized buffer components (Applied Biosystems), primers (SNF1 and SNR1or SNF2 and SNR2) to a final concentration of 0.9 μM, probe to a final concentration of 0.2 μM, and 5 μl of template DNA. Sterile nuclease-free water (Promega, Madison, WI) was used to adjust the volume of each reaction to 25 μl. The analyses were performed in 96-well plates using a 7900HT Fast real-time PCR system instrument (Applied Biosystems) on standard mode. Negative controls containing 5 μl of nuclease-free water instead of DNA were included in each run to detect any DNA cross-contamination. DNA extracted from *S. noxia* served as positive control in each PCR run.

### Internal amplification control

A commercially available TaqMan exogenous Internal Positive Control (IPC; Applied Biosystems) was used to detect PCR inhibition. The reagent kit included 10X Exogenous IPC Primer and Probe (VIC™ Probe) mix, 10X Exogenous IPC Blocking Reagent, and 50X Exogenous IPC DNA. The qPCR assay was optimized to conditions suitable for the detection of both the target organism and the internal positive control; thus, absence or decrease of amplification of the IPC DNA in each multiplex PCR reaction indicated the presence of PCR inhibitors. Several dilutions (i.e., 10^−1^ to 10^−6^) of each DNA sample were tested, to determine and eliminate potential PCR inhibitors. A spectrophotometer with the nanoCell accessory was used as indicated above to demonstrate the presence of DNA in negative non-target PCR samples.

### Data Analysis

Replicate PCR amplifications were performed (n = 4). Once amplification was completed, the data were analyzed and plotted (fluorescence vs. cycle number) using the software provided with the 7900HT PCR instrument. The level of amplification was reported by the software as the mean Cycle threshold (Ct) value of replicate samples. Ct refers to the PCR cycle at which fluorescence (i.e., amplification product) is first detected, and is inversely proportional to the initial DNA template concentration. A Ct value of 40 represents no target DNA present.

## Results

### Primer and probe design

Results from the Protein Family Sorter (PFS) using PATRIC showed 14 unique proteins for *S. noxia*, but the protein BLAST did not find a reliable output to design the primer/probe set in this region. Therefore, the primer sets were designed using the 16S rRNA sequence [GenBank:AF287799]. Both primer sets, primer set 1 (SNF1, SNR1) and primer set 2 (SNF2, SNR2) amplified the target organism. Primer set 1 was found to produce the lowest Ct values, indicating that it was more sensitive at detecting the target organism (Table 3). Therefore, this primer set was selected for additional optimization and validation experiments.

**Table 3:** Quantitative PCR results for *S. noxia* detection

Target organism	Primer set	Dilution factor		
		10^0^	10^−1^	10^−2^
		Mean cycle threshold (Ct) value ± S.E. (n = 2)		
*S. noxia* (ATCC 43541)	1	11.68 (0.56)	14.99 (0.42)	17.97 (0.02)
	2	13.55 (0.95)	15.51 (1.12)	18.21 (0.16)
*S. noxia* (ATCC 51893)	1	9.39 (0.10)	13.56 (0.08)	16.91 (0.11)
	2	11.66 (0.44)	13.35 (0.28)	17.23 (0.11)

### PCR optimization

The nine different combinations of primer concentrations tested produced optimal amplification signal in five of the nine combinations, ranging between 19–20 Ct values for the 0.2 μM/0.9 μM, 0.5/0.5 μM, 0.5/0.9 μM, 0.9/0.5 μM, and 0.9/0.9 μM forward and reverse primer concentrations, respectively (data not shown). Probe optimization results showed that three of the five different concentrations (i.e., 0.15 μM, 0.2 μM, and 0.25 μM) produced the lowest Ct values (<20) (data not shown). As recommended by the instrument and software manufacturer, 0.9 μM forward and reverse primer concentrations, and 0.2 μM probe concentration were selected for specificity testing. The lower detection limit of the *Selenomonas noxia* PCR assay (using *S. noxia* ATCC 51893 as the test organism) was 583 fg/reaction.

### Specificity Testing

Quantitative PCR amplification of the target organism using the primers and probes, reaction conditions, and cycling parameters described above resulted in amplification of the expected 97-bp fragment from the three *S. noxia* strains tested (Table 4). The non-target *Selenomonas* spp. tested did not amplify with the designed primers and probe. The remaining non-target organisms were also not amplified with the 16S qPCR assay developed (Table 4).

**Table 4:** Specificity testing of *S. noxia* qPCR assay

Test organism	ATCC #	PCR results
*Bacillus cereus*	14579	Negative
*Candida albicans*	14053	Negative
*Centipeda periodontii*	35019	Negative
*Klebsiella pneumoniae*	4352	Negative
*Lactobacillus acidophilus*	3456	Negative
*Pectinatus cervisiiphilus*	29359	Negative
*Pseudomonas aeruginosa*	27853	Negative
*Selenomonas artemidis*	43528	Negative
*Selenomonas dianae*	43527	Negative
*Selenomonas flueggei*	43531	Negative
*Selenomonas infelix*	43532	Negative
*Selenomonas noxia*	43541	Positive
*Selenomonas noxia*	51893	Positive
*Selenomonas noxia*	700225	Positive
*Selenomonas sputigena*	35185	Negative
*Staphylococcus aureus*	25923	Negative
*Streptococcus mutans*	25175	Negative

### PCR inhibition

The target gene was amplified in all concentrations of DNA tested from three *S. noxia* strains. However, the IPC PCR demonstrated the presence of PCR inhibition, and these DNA extracts necessitated dilution (10^−4^ to 10^−5^) to remove the inhibitors (Table 5).

**Table 5:** PCR results showing inhibition in *S. noxia* samples

	PCR results (Ct values)				
Sample	Dilution	Sample		IPC	
*S. noxia* (ATCC 43541)					
	10^0^	14.60	15.01	40.00	40.00
	10^−1^	17.76	17.94	40.00	40.00
	10^−2^	21.30	21.23	40.00	40.00
	10^−3^	24.79	24.71	40.00	31.21
	10^−4^	28.13	28.01	28.08	28.11
*S. noxia* (ATCC 51893)					
	10^0^	13.36	13.24	40.00	40.00
	10^−1^	15.34	15.45	40.00	40.00
	10^−2^	19.59	19.26	40.00	40.00
	10^−3^	22.54	23.05	40.00	40.00
	10^−4^	28.29	28.23	40.00	40.00
	10^−5^	30.80	30.77	28.08	28.93
*S. noxia* (ATCC 700225)					
	10^0^	20.90	20.00	40.00	40.00
	10^−1^	nd^1^	nd^1^	nd^1^	nd^1^
	10^−2^	22.92	23.00	40.00	40.00
	10^−3^	26.68	26.48	40.00	40.00
	10^−4^	29.95	30.11	28.53	28.22
	10^−5^	33.53	33.53	27.73	28.06
No template control		40.00	40.00	28.40	28.12

^1^not determined

Dilutions (10^−1^ to 10^−6^) were necessary to remove the inhibitors in non-target DNA extracts (Table 6). In the absence of inhibition, the IPC DNA (i.e., no template control) amplified with a mean Ct value of 29.4 ± 0.3 (standard error; S.E.). It was necessary to dilute all samples, target and non-target DNA, to remove the inhibitors. All non-target DNA extracts produced negative results (Ct = 40) for the *S. noxia* PCR assay even when PCR inhibitors had been removed by sample dilution. These negative non-target PCR samples contained DNA as demonstrated spectrophotometrically with the nanoCell accessory (data not shown).

**Table 6:** Data showing dilution at which inhibition was resolved (n = 2 for all samples, except those in bold which consisted of four replicates)

		PCR results (Mean Ct value)
Sample	Dilution	IPC
*Bacillus cereus*	1.00E + 00	40.00
	1.00E-02	40.00
	1.00E-04	40.00
	1.00E-05	31.43
	**1.00E-06**	**29.48**
*Candida albicans*	1.00E + 00	40.00
	**1.00E-01**	**30.76**
*Centipeda periodontii*	1.00E + 00	40.00
	1.00E-02	40.00
	1.00E-03	40.00
	**1.00E-04**	**29.01**
*Klebsiella pneumonia*	1.00E + 00	40.00
	**1.00E-01**	**30.42**
*Lactobacillus acidophilus*	1.00E + 00	40.00
	1.00E-01	40.00
	1.00E-02	40.00
	**1.00E-03**	**29.84**
*Pseudomonas aeruginosa*	1.00E + 00	40.00
	**1.00E-01**	**30.47**
*Pectinatus cerevisiiphilus*	1.00E + 00	40.00
	1.00E-01	40.00
	1.00E-02	40.00
	**1.00E-03**	**28.70**
*Staphylococcus aureus*	1.00E + 00	40.00
	1.00E-02	40.00
	**1.00E-03**	**29.73**
*Selenomonas artemidis*	1.00E + 00	40.00
	1.00E-01	40.00
	1.00E-02	40.00
	**1.00E-03**	**28.72**
*Selenomonas dianae*	1.00E + 00	40.00
	1.00E-01	40.00
	1.00E-02	40.00
	**1.00E-03**	**28.22**
*Selenomonas flueggeii*	1.00E + 00	40.00
	1.00E-01	40.00
	1.00E-02	40.00
	**1.00E-03**	**28.82**
*Selenomonas infelix*	1.00E + 00	40.00
	1.00E-01	40.00
	**1.00E-02**	**29.32**
*Streptococcus mutans*	1.00E + 00	40.00
	1.00E-01	40.00
	1.00E-02	29.98
	**1.00E-03**	**28.25**
*Selenomonas sputigena*	1.00E + 00	40.00
	1.00E-01	40.00
	1.00E-02	40.00
	**1.00E-03**	**28.56**
Positive control		28.64
Negative control		29.23
No amplification control		40.00

Bold font represents those samples consisting of four replicates

## Discussion

Methods for the detection of *Selenomonas noxia* have been limited to culture and DNA hybridization tests. The exclusion of oxygen at every stage of isolation, transfer and preservation has been documented to be an essential prerequisite of culture analysis methods [16]. The anaerobic culture technique used in the detection of *S. noxia*, and the discrepancy between salivary recovery and subgingival presence have been significant, which makes this approach impractical for use in the microbial diagnosis of periodontitis patients [15]. In this study, a qPCR method for the rapid, specific, and sensitive detection of *S. noxia* was developed and evaluated. The method consists of extraction of DNA prior to a TaqMan-based qPCR amplification, and can be completed within a day. We found that the 16S rRNA was more suitable than the protein coding regions for the identification of a specific detection sequence and the development of the qPCR assay.

The use of specific virulence factors for species level identification has been shown to be effective [17–19]. In this study, the protein families under the phylum Firmicutes were accessed using the Pathosystems Resource Integration Center (PATRIC) computational tool (http://www.patricbrc.org/portal/portal/patric/FIGfam Sorter). Screening for a unique protein family using PATRIC was done for *S. noxia*, because specific virulence factors for this organism have not been published. The selected protein BLAST search to locate unique proteins (e.g., the conserved hypothetical protein, GenBank: ZP06602469; sucC protein, GenBank: ZP06602580) did not show an acceptable output to proceed with primer/probe design. The underlying reasons were not clear, but may be attributed to the incomplete genome sequence/annotation of *S. noxia* in the NCBI GenBank database.

The 16S rRNA gene qualifies as the most comprehensive single gene database that can be used to classify bacteria phylogenetically. Although much of the 16S rRNA is highly conserved among many bacterial families, portions of the gene are unique and can be used to speciate bacteria [20, 21]. Bacterial 16S rRNA genes generally contain nine hypervariable regions that demonstrate considerable sequence diversity among different bacterial species and can be used for species identification [22]. The finding by Chakravorty *et al.* [23] suggested that the V4-V8 region has a higher degree of conserved sequences compared to other hypervariable regions for species level identification. In contrast, *S. noxia* has hypervariable sequences in the V3, V4, V6 and V8 regions. In this study, the primers and probes were designed to amplify a 97 bp sequence in the V8 region of the 16S rRNA.

**Figure 1:**
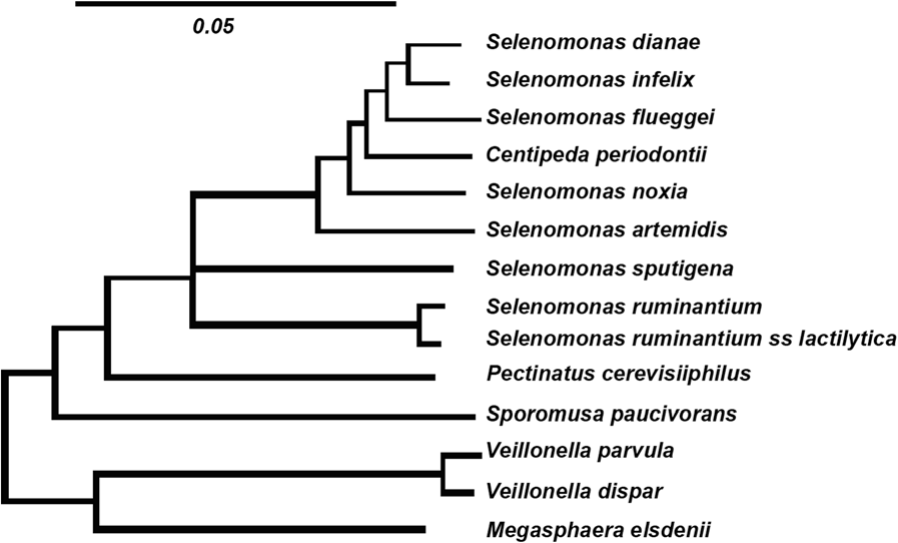
Phylogenetic tree for the Selenomonads and related bacteria. The scale indicates a 5 % difference in nucleotide sequence, as determined by taking the sum of all branch lengths connecting two species [30]

The phylogeny of species of *Selenomonas* and related bacteria has been determined by using 16S rRNA sequence analysis [24]. The *Selenomonas* group is phylogenetically coherent (Figure 1) with interspecies homology levels of 90 to 99 %. *Selenomonas dianae*, *S. infelix*, *S. flueggei*, *S. noxia*, *S. artemidis*, and *Centipeda periodontii* form a very tight cluster with a homology range of 96 to 99 %. *Selenomonas sputigena* and *S. ruminantium* have an average sequence homology of 94 % with members of this cluster [25].

The PCR assay described in this study was designed to detect and amplify a sequence on the 16S rRNA gene specific to *S. noxia*. The probes designed in similar studies were designed to amplify conserved regions common to most oral bacteria in the 16S rRNA [8, 9]. The results from the selectivity study presented here showed that the selected primers and probe provided specific detection of *S. noxia*, with 100 % specificity. To properly validate a PCR assay, it must be demonstrated that the designed primers and probes amplify only the target species and do not cross-react with non-target organisms. A negative result from a non-target sample may be a false negative, if no DNA is present or PCR inhibitors are present. In this study, we used a spectrophotometer with the nanoCell accessory to demonstrate the presence of DNA in negative non-target PCR samples. Previous research has shown that PCR inhibitors can co-extract with DNA samples [26, 27], and similarly our samples showed complete inhibition of the IPC DNA, and necessitated a 10^−6^ dilution to remove all PCR inhibitors. However, the PCR assay was able to detect the target gene at all DNA concentrations tested despite inhibition of the IPC. The reason for this observation is unknown.

Comparison of PCR with other molecular biology techniques used for the detection of pathogenic bacteria showed that the selection of a test depends on the specific organism of interest [28, 29]. In a study conducted by Siqueira *et al.* [30], the PCR method yielded the highest number of positive results for most target species when compared with checkerboard DNA–DNA hybridization for the detection of endodontic pathogens. Future research should expand the validation of the primers and probe designed and optimized against other oral pathogens, more *S. noxia* strains, and other *Selenomonas* species (including *S. ruminantium*) to confirm the specificity of the assay.

## Conclusions

The 16S qPCR assay designed in this study was used to specifically amplify *S. noxia*. This assay will be of value in the clinical setting and for future research involving the enhanced detection of *S. noxia*. The test can be used for epidemiological studies in understanding the role of *S. noxia* in disease processes including, but not limited to, oral health and infectobesity.

## Abbreviations

ATCC: American Type Culture Collection
BLAST: Basic Local Alignment Search Tool
CP: Chronic periodontitis
Ct: cycle threshold
FISH: fluorescence *in-situ* hybridization
GAP: Generalized aggressive periodontitis
HLO: *Helicobacter*-Like Organism
IPC: Internal positive control
PATRIC: Pathosystems Resource Integration Center
PCR: Polymerase chain reaction
PFS: Protein Family Sorter
qPCR: quantitative PCR
S.E.: Standard Error
TE: TRIS-EDTA
UNG: uracil-N-glycosylase

## References

[1] Dobell C (1958). Antony van Leeuwenhoek and his "Little animals".

[2] Craig RG, Boylan R, Yip J, Bamgboye P, Koutsoukos J, Mijares D (2001). Prevalence and risk indicators for destructive periodontal diseases in 3 urban American minority populations. J Clin Periodontol.

[3] Tanner A, Malden MFJ, Macuch PJ, Murray LL, Kent RL (1998). Microbiota of health, gingivitis, and initial periodontitis. J Clin Periodontol.

[4] Torresyap G, Haffajee AD, Uzel NG, Socransky SS (2003). Relationship between periodontal pocket sulfide levels and subgingival species. J Clin Periodontol.

[5] Moore L, Johnson J, Moore W. *Selenomonas noxia* sp. *nov., Selenomonas flueggei* sp. *nov., Selenomonas infelix* sp. *nov., Selenomonas dianae* sp. *nov*., and *Selenomonas artemidis* sp. *nov*. from the Human Gingival Crevice. International Journal of Systemic Bacteriology. 1987;36(3):271–80.

[6] Riep B, Edesi-Neuss L, Claessen F, Skarabis H, Ehmke B, Flemmig TF (2009). Are putative periodontal pathogens reliable diagnostic markers?. J Clin Microbiol.

[7] Tanner AC (2015). Anaerobic culture to detect periodontal and caries pathogens. J Oral Biosci.

[8] Faveri M, Mayer MP, Feres M, de Figueiredo LC, Dewhirst FE, Paster BJ (2008). Microbiological diversity of generalized aggressive periodontitis by 16S rRNA clonal analysis. Oral Microbiol Immunol.

[9] Drescher J, Schlafer S, Schaudinn C, Riep B, Neumann K, Friedmann A (2010). Molecular epidemiology and spatial distribution of *Selenomonas* spp. in subgingival biofilms. Eur J Oral Sci.

[10] Kumar PS, Griffen AL, Moeschberger ML, Leys EJ (2005). Identification of candidate periodontal pathogens and beneficial species by quantitative 16S clonal analysis. J Clin Microbiol.

[11] Haffajee AD, Cugini MA, Tanner A, Pollack RP, Smith C, Kent RL (1998). Subgingival microbiota in healthy, well-maintained elder and *periodontitis* subjects. J Clin Periodontol.

[12] Andersen LP, Lange P, Tvede M (2010). *Selenomonas* may puzzle the diagnosis of *Helicobacter pylori* in gastric mucosa. Eur J Clin Microbiol Infect Dis.

[13] Dahlén G, Manji F, Baelum V, Fejerskov O (1992). Putative periodontopathogens in "diseased" and "non-diseased" persons exhibiting poor oral hygiene. J Clin Periodontol.

[14] Goodson JM, Groppo D, Halem S, Carpino E (2009). Is Obesity an Oral Bacterial Disease?. J Dent Res.

[15] Boutaga K, Savelkoul P, Winkel E, van Winkelhoff A (2007). Comparison of Subgingival Bacterial Sampling with Oral Lavage for Detection and Quantification of Periodontal Pathogens by Real-Time Polymerase Chain Reaction. J Periodontol.

[16] Shouche Y, Dighe A, Dhotre D, Patole M, Ranade D (2009). The genus *Selenomonas*. Bergey's Manual of Systematic Bacteriology.

[17] Meng J, Zhao S, Doyle MP, Mitchell SE, Kresovich S (1997). A multiplex PCR for identifying Shiga-like toxin-producing *Escherichia coli* O157:H7. Lett Appl Microbiol.

[18] Fratamico PM, Sackitey SK, Wiedmann M, Deng MY (1995). Detection of *Escherichia coli O157:H7* by multiplex PCR. J Clin Microbiol.

[19] Wang G, Clark C, Rodgers F. Detection in *Escherichia coli* of the genes encoding the major virulence factors, the genes defining the O157:H7 serotype, and components of the type 2 Shiga toxin family by multiplex PCR. J Clin Microbiol. 2002;3613–3619. 10.1128/JCM.40.10.3613-3619.2002 PMC130888 12354854

[20] Clarridge JE (2004). Impact of 16S rRNA gene sequence analysis for identification of bacteria on clinical microbiology and infectious diseases. Clin Microbiol Rev.

[21] Petti CA, Polage CR, Schreckenberger P (2005). The role of 16S rRNA gene sequencing in identification of microorganisms misidentified by conventional methods. J Clin Microbiol.

[22] Van de Peer Y, Chapelle S, De Wachter R (1996). A quantitative map of nucleotide substitution rates in bacterial rRNA. Nucleic Acids Res.

[23] Chakravorty S, Helb D, Burday M, Connell N, Alland D (2007). A detailed analysis of 16S ribosomal RNA gene segments for the diagnosis of pathogenic bacteria. J Microbiol Methods.

[24] Dewhirst FE, Tuste C, Izard J, Paster B, Tanner ACR, Wen-Han Y (2010). **The Human Oral Microbiome**. J. Bacteriol..

[25] Hespell R, Paster B, Dewhirst F (2006). The Genus *Selenomonas*. Prokaryotes.

[26] Cruz-Perez P, Buttner MP, Stetzenbach LD (2001). Specific detection of *Stachybotrys chartarum* in pure culture using quantitative polymerase chain reaction. Molecular Cellular Probes.

[27] Grevelding CG, Kampkotter A, Hollmann M, Schafer U, Kunz W (1996). Direct PCR on fruitflies and blood flukes without prior DNA isolation. Nucleic Acids Res.

[28] Kimura H, Morita M, Yabuta Y, Kuzushima K, Kato K, Kojima S (1999). Quantitative Analysis of Epstein-Barr Virus Load by Using a Real-Time PCR Assay. J Clin Microbiol.

[29] Scaletsky I, Fabbricotti S, Aranda K, Morais M, Fagundes-Neto U (2002). Comparison of DNA Hybridization and PCR Assays for Detection of Putative Pathogenic Enteroadherent *Escherichia coli*. J Clin Microbiol.

[30] Siqueira J, Rôças I, De Uzeda M, Colombo AP, Santos K (2002). Comparison of 16S rDNA-based PCR and checkerboard DNA–DNA hybridisation for detection of selected endodontic pathogens. J Med Microbiol.

[31] Brosius J, Palmer ML, Kennedy PJ, Noller HF (1978). Complete nucleotide sequence of a 16S ribosomal RNA gene from *Escherichia coli*. Proc Natl Acad Sci.

